# Vitamin D as Modulator of Drug Concentrations: A Study on Two Italian Cohorts of People Living with HIV Administered with Efavirenz

**DOI:** 10.3390/nu13103571

**Published:** 2021-10-12

**Authors:** Jessica Cusato, Massimo Tempestilli, Andrea Calcagno, Alessandra Vergori, Pierluca Piselli, Miriam Antonucci, Valeria Avataneo, Alice Palermiti, Stefania Notari, Andrea Antinori, Giovanni Di Perri, Chiara Agrati, Antonio D’Avolio

**Affiliations:** 1 Laboratory of Clinical Pharmacology and Pharmacogenetics, Department of Medical Sciences, Amedeo di Savoia Hospital, University of Turin, 10149 Turin, Italy; miriam.antonucci20@gmail.com (M.A.); valeria.avataneo@unito.it (V.A.); alice.palermiti@unito.it (A.P.); antonio.davolio@unito.it (A.D.); 2 National Institute for Infectious Diseases Lazzaro Spallanzani IRCCS, 00149 Rome, Italy; massimo.tempestilli@inmi.it (M.T.); alessandra.vergori@inmi.it (A.V.); pierluca.piselli@inmi.it (P.P.); stefania.notari@inmi.it (S.N.); andrea.antinori@inmi.it (A.A.); chiara.agrati@inmi.it (C.A.); 3 Unit of Infectious Diseases, Department of Medical Sciences, Amedeo di Savoia Hospital, University of Turin, 10149 Turin, Italy; andrea.calcagno@unito.it (A.C.); giovanni.diperri@unito.it (G.D.P.)

**Keywords:** plasma concentrations, vitamin D, seasonality, drug metabolism

## Abstract

To date, vitamin D seems to have a significant role in affecting the prevention and immunomodulation in COVID-19 disease. Nevertheless, it is important to highlight that this pro-hormone has other several activities, such as affecting drug concentrations, since it regulates the expression of cytochrome P450 (CYP) genes. Efavirenz (EFV) pharmacokinetics is influenced by CYPs, but no data are available in the literature concerning the association among vitamin D levels, seasonality (which affects vitamin D concentrations) and EFV plasma levels. For this reason, the aim of this study was to evaluate the effect of 25-hydroxy vitamin D (25(OH)D3) levels on EFV plasma concentrations in different seasons. We quantified 25(OH)D3 by using chemiluminescence immunoassay, whereas EFV plasma concentrations were quantified with the HPLC–PDA method. A total of 316 patients were enrolled in Turin and Rome. Overall, 25(OH)D3levels resulted in being inversely correlated with EFV concentrations. Some patients with EFV levels higher than 4000 ng/mL showed a deficient 25(OH)D3 concentration in Turin and Rome cohorts and together. EFV concentrations were different in patients without vitamin D supplementation, whereas, for vitamin D-administered individuals, no difference in EFV exposure was present. Concerning seasonality, EFV concentrations were associated with 25(OH)D3 deficiency only in winter and in spring, whereas a significant influence was highlighted for 25(OH)D3 stratification for deficient, insufficient and sufficient values in winter, spring and summer. A strong and inverse association between 25(OH)D3and EFV plasma concentrations was suggested. These data suggest that vitamin D is able to affect drug exposure in different seasons; thus, the achievement of the clinical outcome could be improved by also considering this pro-hormone.

## 1. Introduction

Vitamin D is recognized as a prohormone. Vitamin D is classified as a nutrient, and it is also synthesized by the human body through the action of sunlight. These dual sources of vitamin D make it challenging to develop dietary reference intake values [1].

Worldwide, vitamin D deficiency represents a public health problem in all age groups; however, studies are still lacking in most countries, particularly on risk groups. Data recorded by the National Health and Nutrition Examination Survey (NHANES) show that 79% of the elderly adult population has vitamin D deficiency or insufficiency [2,3]. This phenomenon seems to be frequent in Italy among elderly adults and, particularly, during winter months [4,5]. Different pathologies have been associated with vitamin D deficiency, including COVID-19 [6].

Ultraviolet light is responsible for vitamin D synthesis in skin; cholecalciferol is hydroxylated to calcifediol (25-hydroxy vitamin D, 25(OH)D3) in the liver through cytochrome P-450 (CYP) 27A1 and CYP2R1 and, in the kidney, calcitriol (1,25-dihydroxy vitamin D, 1,25(OH)D3) is synthesized through CYP27B1. Then 1,25-VD is transported in the bloodstream through the vitamin-D-binding protein (VDBP).

In HIV-positive patients, reduced vitamin D concentrations were often found at various levels of severity and have been linked to low bone-mineral density and related disorders, subclinical vascular disease, kidney function decline, endocrine disorders, liver fibrosis, preterm delivery and neurocognitive impairment [7,8,9,10,11,12].

Vitamin D modulates the expression of many genes through its receptor (vitamin D receptor, VDR); particularly, it has an impact on the expression of gene-encoding transporters and enzymes responsible for drug transport and metabolism, such as CYPs [13].

Drocourt et al. showed that vitamin D induces *CYP3A4* and, to a lesser extent, *CYP2B6* and *CYP2C9* genes expression in human hepatocytes [14].

Several drugs are metabolized by CYP3A4; its gene shows vitamin D responsive elements (VDRE), and its expression is upregulated in the presence of 1,25(OH)D3. Consequently, vitamin D may alter CYP3A4-metabolized drugs’ concentrations, as shown for immunosuppressants: Lindh et al. suggested tacrolimus and sirolimus seasonal variability according to changes in vitamin D levels (which depends on sunlight exposure); they observed lower drug concentration in July to September than in January to March [15]. In addition, vitamin D is able to affect CYP2B6 gene expression; consequently, this enzyme metabolized drugs, for example, Efavirenz (EFV).

Vitamin D may interact with several drugs, potentially altering drug toxicity or efficacy, but also drugs may affect vitamin D metabolism and status [16]. In fact, the 25-hydroxylase CYP3A4, which is a phase 1 biotransformation enzyme for several drugs, as suggested before, is able to convert precursors to 25(OH)D3.

Moreover, antiretroviral drugs are pregnane X receptor (PXR) ligands; thus, they are able to activate it and the related pathway [16]. PXR is important when considering xenobiotics and drugs detoxifications; it is able to bind to VDRE, affecting the expression of genes normally regulated by vitamin D. In fact, 24-hydroxylases and other CYPs resulted in being upregulated in the presence of PXR.

Studies reported that in vitro HIV protease inhibitors, particularly ritonavir, inhibit the conversion of 25(OH)D3 to 1,25(OH)D3 and 1,25(OH)D3 degradation [17].

EFV pharmacokinetic exposure shows high inter-patient variability, and it is related to toxicity in terms of neurological problems: Burger et al. analyzed 255 individuals, suggesting 48 (18.9%) patients had EFV toxic concentrations [18]. Furthermore, they highlighted gender and race as important factors determining inter-patient EFV plasma-level variability. In conclusion, they recommended physicians to pay particular attention to females and non-caucasian ethnicity patients, since they are more predisposed to EFV-induced toxicity. Consequently, it could be useful to evaluate which factors are able to affect EFV exposure, particularly considering that vitamin D seems to influence the expression of CYPs involved in this drug metabolism.

Not many data are available in the literature concerning the association between EFV and 25(OH)D3 levels in Italian patients. Moreover, in clinical practice, vitamin D’s use as supplements to prevent and treat a wide range of clinical conditions has increased substantially over the last decade in people living with HIV (PLWH), even in different geographical latitudes.

For these reasons, the aim of this study was to analyze EFV and vitamin D relationship in two cohorts, from Turin (North of Italy) and from Rome (Center of Italy), consisting of HIV-positive patients seen for care, in order to evaluate vitamin D’s effect on EFV exposure in different seasons. An association between 25(OH)D3 and EFV plasma concentrations was suggested.

## 2. Materials and Methods

### 2.1. Study Design

A retrospective cohort study was performed in PLWH treated at Amedeo di Savoia (Turin, Italy) and National Institute for Infectious Diseases “L. Spallanzani”, IRCCS (Rome, Italy) between January 2015 and January 2018.

Inclusion criteria were age ≥ 18 years, good general condition (without other diseases), on EFV-containing therapy for >7 days, absence of any interacting drugs (such as rifampicin, methadone or erythromycin), no co-infection, drug intake 12 (±3) h before blood withdrawal and reported medication adherence above 90% (Ethic Committee approvals: COVID study 53/2018 for Rome and CS2/325 del 8/8/2017 for Turin).

For each patient, the following data were recorded: demographics (e.g., sex and age); HIV stage (according to the Centers for Disease Control and Prevention (CDC)) estimation of adherence according to the proportion of visits “on time” (proportion of visits respecting the deadline given by appointment compared with the total visits); start of first-line therapy; symptoms; diseases and/or concomitant medications at the time of the visit; antiretroviral therapy in progress; time and date of the last administration of antiretroviral drugs.

### 2.2. Efavirenz Plasma Concentrations

Sampling was performed the day after the evening dose of EFV (12 ± 3 h). Plasma samples were obtained from a lithium–heparin tube (7 mL) and were stored in cryovials at −20 °C before analysis.

EFV concentrations at 12 h after intake (C12) were determined by a high-performance liquid chromatography (HPLC) system coupled with a photodiode array (PDA), using solid-phase extraction for frozen plasma samples, according to a previously described and fully validated method [19]. Patients with undetectable concentrations were considered non-adherent and were excluded from the analyses. All patients with EFV exposure higher than the lower limit of quantification were considered eligible for the analysis. EFV C12 therapeutic range is within 1000–4000 ng/mL [20].

### 2.3. Quantification of 25-Hydroxyvitamin D 

Contextually to EFV quantification, total serum 25(OH)D3 was quantified by using a chemiluminescence immunoassay (CLIA; DiaSorin LIAISON^®^ 25 OH Vitamin D TOTAL Assay. This method does not allow for us to differentiate between D2 and D3 forms.

Serum Vitamin D levels were classified, according to manufacture reference values, on (i) deficiency (≤10 ng/mL), (ii) insufficiency (11 to 30 ng/mL) and (iii) sufficiency (>30 ng/mL) [21].

### 2.4. Statistical Analysis

All of the continuous variables were tested for normality with the Shapiro–Wilk test. The Kolmogorov–Smirnov test was performed in order to evaluate the distribution, comparing a sample with a reference probability distribution. Non-normally distributed variables were described as median and interquartile range. The correlation between continuous variables was performed by parametric and non-parametric tests (Pearson and Spearman). Non-normal variables were resumed as median values and interquartile range (IQR), whereas categorical variables were resumed as numbers with percentages. Kruskal–Wallis and Mann–Whitney analyses were considered for differences in continuous variables between different groups (such as vitamin D levels stratification and seasons), considering a statistical significance with a two-sided *p*-value < 0.05. Chi-squared test was used to evaluate differences between categorical variables (such as vitamin D stratification values and EFV-associated cutoff values).

All of the tests were performed with IBM SPSS Statistics for Windows v.26.0 (IBM Corp., Chicago, IL, USA).

## 3. Results

### 3.1. Patients Characteristics

Characteristics of the 316 analyzed patients are reported in Table 1: 227 patients were enrolled in Turin, whereas 89 individuals were enrolled in Rome.

### 3.2. Vitamin D Distribution

The 25(OH)D3 levels distribution (≤10, 11–30 and >30 ng/mL) was reported in Table 1; viral loads for the Rome center were not available, since these data were difficult to obtain after years. Overall, the 25(OH)D3 concentrations were not significantly different in the two cohorts (*p* = 0.657), and in both cohorts, a similar frequency of patients presenting 25(OH)D3 level below 30 ng/mL (deficiency 12.4% vs. 10.1%; insufficiency 68.5% vs. 63.0%) was observed. Furthermore, an increased number of patients had 25(OH)D3 concentrations higher than 30 ng/mL (26.9% vs. 19.1%) in the Turin cohort, but without being statistically significance.

### 3.3. Efavirenz Distribution According to Vitamin D Levels

Of note, 25(OH)D3 levels resulted in being inversely correlated with EFV concentrations (r^2^ = 0.016; *p* = 0.020, Supplementary Material Figure S1).

**Figure 1:**
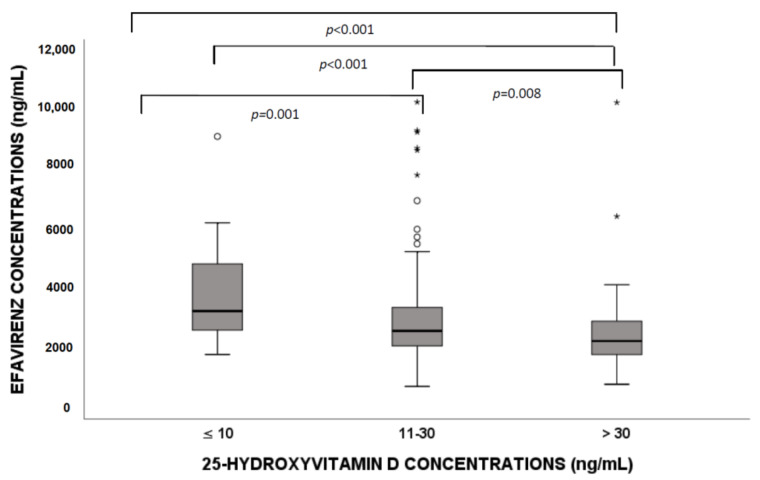
Efavirenz exposure according to 25-hydroxyvitamin D (25(OH)D3) level stratification (deficiency, insufficiency and sufficiency). Circles and stars indicate “out” values (small circle) and “far out” values (star).

When comparing HIV patients with different 25(OH)D3 levels, we found that significant differences in EFV concentration (deficiency vs. insufficiency, *p* = 0.001; deficiency vs. sufficiency, *p* < 0.001; insufficiency vs. sufficiency, *p* = 0.008; Figure 1) were suggested. In particular, higher drug concentrations in patients with 25(OH)D3 deficiency were highlighted.

A possible association between 25(OH)D3 levels and EFV-associated toxicity by defining a 4000 ng/mL cutoff for EFV concentration was considered [20]: a significant higher proportion of patients with EFV levels higher than 4000 ng/mL showed a deficiency in 25(OH)D3 concentration in Turin (*p* = 0.017) and Rome (*p* < 0.001) cohorts and together (*p* < 0.001) (see Table 2).

Patients were supplemented with vitamin D only in the Turin cohort. In Table 3, patients were divided in supplemented or not, and then, for both groups, 25(OH)D3 stratification for deficient, insufficient and sufficient values was considered. EFV concentrations were statistically different (*p* = 0.042) in patients without vitamin D supplementation, whereas, for vitamin D-administered individuals, no deficient patients were present; in addition, they did not show a statistical significant difference (*p* = 0.622).

### 3.4. Seasonality

Concerning seasonality, EFV concentrations were associated with vitamin D deficiency (≤10 ng/mL) only in winter (*p* = 0.001, deficient patients = 11/88) and in spring (*p* = 0.017, deficient patients = 12/82), but not in summer (*p* = 0.149, deficient patients = 1/66) and autumn (*p* = 0.494, deficient patients = 10/80).

A statistical significance was highlighted for 25(OH)D3 stratification in winter (*p* = 0.002), spring (*p* = 0.039) and summer (*p* = 0.011), but not for autumn (*p* = 0.391).

## 4. Discussion

Several studies have shown that vitamin D is able to affect drug concentrations [22,23,24,25] and clinic features [26,27,28,29]. In this context, recently, our group’s work (accepted for publication) focused on the seasonality of other antiretroviral drugs, showing a trend in concentrations during the year, especially for etravirine, maraviroc and lopinavir [30].

In this study, 316 PLWH treated with EFV were included. EFV and 25(OH)D3 concentrations were investigated: most of patients had 25(OH)D3 deficiency or insufficiency, as shown in Table 1. This seems to agree with percentages evidenced by Cervero et al., who analyzed a cohort of 352 HIV-infected individuals: deficiency was present in 44%, whereas insufficiency was present in 71.6% [31]. These data are related to patients living in Spain, which has a similar latitude to Italy. 

Furthermore, in this study, an inverse correlation between 25(OH)D3 levels and EFV exposure was demonstrated according to what shown by Lindh et al.; in fact, tacrolimus and sirolimus immunosuppressant agents’ concentrations decreased with an increased vitamin D level. This could be due to vitamin D ’s inductive effect on genes encoding for protein involved in these drugs’ metabolism and excretion (CYP3A5, CYP2B6 and ABCB1 genes encoding for CYP3A5, CYP2B6 enzymes and for P-glycoprotein transporter) [13,15]. In addition, as shown for tacrolimus and sirolimus, as well as for EFV, seasonality could have an impact in terms of EFV plasma variation.

A possible interaction between 25(OH)D3 and antiretrovirals has been evidenced for other anti-HIV drugs; for example, tenofovir disoproxil fumarate (TDF) is an anti-HIV drug which causes bone, endocrine and renal changes, but mechanisms are not well described [32]. In a cohort of 118 patients taking TDF, the authors suggested that the highest quintile of TDF plasma concentrations was associated with increased VDBP, 25(OH)D3 and calcium, but lower 1,25(OH)D3. Furthermore, higher plasma TDF exposure was related to increased VDBP and lower 1,25(OH)D3, suggesting a functional vitamin D deficiency explaining TDF-associated higher parathyroid hormone levels [33].

In the Turin cohort, most of patients showed 25(OH)D3 concentrations higher than 30 ng/mL and, generally, increased 25(OH)D3 levels: this could be in contrast with the considered latitude, since Rome is much closer to the equator (latitude about 41°) compared to Turin (latitude about 45°).

However, due to personal sun-exposure behaviors, professional and outdoor activities, the personal ultraviolet (UV) exposure may be low to negligible if an individual does not engage in outdoor activities. Similarly, an individual may live at a higher latitude, with lower ambient UV levels and with a greater outdoor activity, resulting in a higher personal UV exposure [34].

Moreover, these high levels in the Turin cohort could be explained by the fact that patients are supplemented only in Turin and not in Rome.

Furthermore, considering the EFV cutoff value associated with side effects, a small number of patients had 25(OH)D3 deficiency in EFV concentrations <4000 ng/mL patients compared to the higher percentage in ≥4000 ng/mL ones, confirming vitamin D’s protective role in terms of toxicity, as shown for other kind of pathologies [35,36].

The relationship between vitamin D and EFV exposure could be explained by EFV metabolism by CYP2B6 and vitamin D (particularly 1,25(OH)D3, the active metabolite) that induces the expression of several genes, such as *CYP3A4* and, to a lesser extent, *CYP2B6* and *CYP2C9* ones, in normal differentiated primary human hepatocytes.

This is the first study reporting vitamin D influence on EFV concentrations in two Italian cohorts of HIV-affected patients; particularly, 25(OH)D3 deficiency (≤10 ng/mL) was associated with higher EFV exposure, with a potential risk of adverse effects. Considering EFV neurotoxicity, even at subclinical levels, this may be relevant: it should be highlighted that, in countries with limited resource, EFV is still widely used.

Hypovitaminosis D is present in several clinical conditions, such as diabetes, cancer or HIV infection, in which prevalence varies from 14% to 52% depending on gender, lifestyle, season, ethnicity, geographic position and type of antiretrovirals [37,38]. Furthermore, a recent analysis showed that vitamin D -deficient HIV-infected patients have an increased risk of having neurocognitive impairment, particularly HIV-associated neurocognitive deficit (HAND), which is associated with EFV therapy, also in asymptomatic patients [39,40,41]. Consequently, for these reasons, it could be very important to conduct vitamin D and drug concentration evaluation during therapy in order to avoid vitamin D and EFV (and other drugs) levels predisposing therapy-associated side effects, such as neurocognitive disorders.

This is the first study in this field, but it has some limitations, such as a lack of data on 1,25(OH)D3 and seasonality, but also on EFV toxicity. It would also be useful to take into consideration other drugs metabolized or transported by enzymes and transporters for which genes’ expressions are affected by vitamin D.

## 5. Conclusions

In conclusion, this manuscript suggests the association between vitamin D levels and EFV exposure in two different cohorts of Italian (Rome and Turin) HIV-affected patients, considering their different latitudes. This study highlights the possible role of vitamin D in predicting EFV levels, despite its reduced use, but it could be useful in order to clarify the involvement of this pro-hormone in affecting other drug concentrations.

Finally, other studies are mandatory in order to better define the role of vitamin D metabolic effects on drugs and their toxicity and to evaluate the possible clinical impact of these findings.

## Figures and Tables

**Table 1:** Patients’ characteristics. “/” indicates no available data.

Characteristics	Turin Cohort	Rome Cohort	Total	*p*-Value
n° patients	227	89	316	
Age (year), median (IQR)	46 (39–51)	45 (37.5–53)	44 (37.5–49)	0.867
Caucasian ethnicity, n (%)	184 (81.1)	72 (80.9)	256 (81)	0.003
Male sex, n (%)	177 (78)	85 (95.5)	262 (82.9)	<0.001
Viral load (copies/mL), median (IQR)	75.5 (28.8–34.8)	/	75.5 (28.8–34.8)	/
CD4 (cells/mL), median (IQR)	717 (553.3–870.0)	546 (408.5–685.5)	584 (450–746)	<0.001
Vitamin D levels (ng/mL), median (IQR)	22.3 (15.1–31.2)	21.9 (16.1–28.8)	22.3 (15.5–30.3)	0.657
Deficiency (≤10 ng/mL), n (%)	23 (10.1)	11 (12.4)	34 (10.8)	0.565
Insufficiency (11–30 ng/mL), n *(*%)	143 (63)	61 (68.5)	204 (64.6)	0.333
Sufficiency (>30 ng/mL), n (%)	61 (26.9)	17 (19.1)	78 (24.7)	0.339
Vitamin D supplementation, n (%)	17 (7.85)	/	17 (7.5)	/

**Table 2:** Efavirenz exposure stratification (< or ≥4000 ng/mL) in deficient, insufficient and sufficient values of vitamin D in the two different cohorts and both together. The *p*-values are obtained through chi squared test (crosstabs).

		Efavirenz < 4000 ng/mL n (%)	Efavirenz ≥ 4000 ng/mL n (%)	Total n (%)	
Turin	Deficiency (≤10)	16 (69.6)	7 (30.4)	23 (100)	*p* = 0.017
	Insufficiency (11–30)	123 (86.0)	20 (14.0)	143 (100)	
	Sufficiency (>30)	57 (93.4)	4 (6.6)	61 (100)	
	Total	196 (86.3)	31 (13.7)	227 (100)	
Rome	Deficiency (≤10)	3 (27.3)	8 (72.7)	11 (100)	*p* < 0.001
	Insufficiency (11–30)	56 (91.8)	5 (8.2)	61 (100)	
	Sufficiency (>30)	17 (100)		17 (100)	
	Total	76 (85.4)	13 (14.6)	89 (100)	
Total	Deficiency (≤10)	19 (55.9)	15 (44.1)	34 (100)	*p* < 0.001
	Insufficiency (11–30)	179 (87.7)	25 (12.3)	204 (100)	
	Sufficiency (>30)	74 (94.9)	4 (5.1)	78 (100)	
	Total	272 (86.1)	44 (13.9)	316 (100)	

**Table 3:** Efavirenz levels according to vitamin D supplementation in the Turin cohort. The *p*-values are obtained through chi squared test (crosstabs).

		Efv Levels < 1000 ng/mL	Efv Levels 1000–4000 ng/mL	Efv Levels > 4000 ng/mL	Total	
No vitamin D supplementation	Deficient (≤10)		16 (69.6)	7 (30.4)	23 (100)	
	Insufficient (11–30)	5 (3.8)	110 (83.3)	17 (12.9)	132 (100)	*p* = 0.042
	Normal (>30)	3 (5.5)	49 (89.1)	3 (5.5)	55 (100)	
	Total	8 (3.8)	175 (83.3)	27 (12.9)	210 (100)	
Vitamin D supplementation	Insufficient (11–30)		8 (72.7)	3 (27.3)	11 (100)	
	Normal (>30)		5 (83.3)	1 (16.7)	6 (100)	*p* = 0.622
	Total		13 (76.5)	4 (23.5)	17 (100)	
All	Deficient (≤10)		16 (69.6)	7 (30.4)	23 (100)	
	Insufficient (11–30)	5 (3.5)	118 (82.5)	20 (14.0)	143 (100)	*p* = 0.064
	Normal (>30)	3 (4.9)	54 (88.5)	4 (6.6)	61 (100)	
	Total	8 (3.5)	188 (82.8)	31 (13.7)	227 (100)	

## Data Availability

Data are available within the text. Patients’ data are available upon request, due to privacy and ethical restrictions.

## Supplementary Materials

The following are available online at https://www.mdpi.com/article/10.3390/nu13103571/s1, Figure S1: Scatter plot of Efavirenz exposure and vitamin D levels with its fit line.
