# Supplementary material for: Vitamin D as Modulator of Drug Concentrations: A Study on Two Italian Cohorts of People Living with HIV Administered with Efavirenz
Source: Nutrients. 2021 Oct 12;13(10):3571. doi: 10.3390/nu13103571 (PMC8538640; doi:10.3390/nu13103571)

Supplementary file

Figure S1: Scatter plot of Efavirenz exposure and vitamin D levels with its fit line.

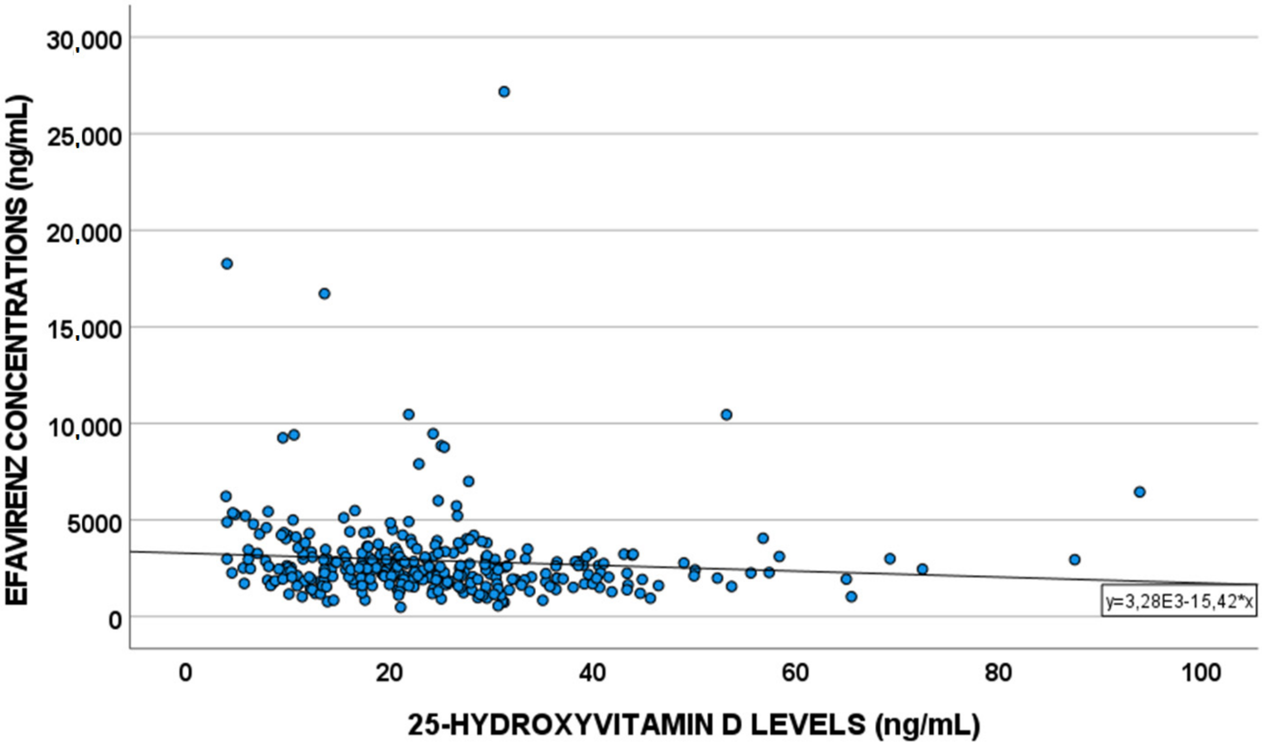

Supplement: Supplementary file 1 [file nutrients-13-03571-s001.zip › nutrients-1390987-supplementary.pdf]
